# Machine Learning Based Computational Gene Selection Models: A Survey, Performance Evaluation, Open Issues, and Future Research Directions

**DOI:** 10.3389/fgene.2020.603808

**Published:** 2020-12-10

**Authors:** Nivedhitha Mahendran, P. M. Durai Raj Vincent, Kathiravan Srinivasan, Chuan-Yu Chang

**Affiliations:** ^1^School of Information Technology and Engineering, Vellore Institute of Technology, Vellore, India; ^2^Department of Computer Science and Information Engineering, National Yunlin University of Science and Technology, Douliu, Taiwan

**Keywords:** gene selection, machine learning, microarray gene expression, supervised gene selection, unsupervised gene selection

## Abstract

Gene Expression is the process of determining the physical characteristics of living beings by generating the necessary proteins. Gene Expression takes place in two steps, translation and transcription. It is the flow of information from DNA to RNA with enzymes’ help, and the end product is proteins and other biochemical molecules. Many technologies can capture Gene Expression from the DNA or RNA. One such technique is Microarray DNA. Other than being expensive, the main issue with Microarray DNA is that it generates high-dimensional data with minimal sample size. The issue in handling such a heavyweight dataset is that the learning model will be over-fitted. This problem should be addressed by reducing the dimension of the data source to a considerable amount. In recent years, Machine Learning has gained popularity in the field of genomic studies. In the literature, many Machine Learning-based Gene Selection approaches have been discussed, which were proposed to improve dimensionality reduction precision. This paper does an extensive review of the various works done on Machine Learning-based gene selection in recent years, along with its performance analysis. The study categorizes various feature selection algorithms under Supervised, Unsupervised, and Semi-supervised learning. The works done in recent years to reduce the features for diagnosing tumors are discussed in detail. Furthermore, the performance of several discussed methods in the literature is analyzed. This study also lists out and briefly discusses the open issues in handling the high-dimension and less sample size data.

## Introduction

Deoxy-ribonucleic Acid (DNA) is a hereditary material containing the genetic information, usually found in the cell’s nucleus. The information inside the DNA is made up of a code consisting of four bases, namely, Adenine, Guanine, Cytosine, and Thymine. Adenine pairs with Thymine and Cytosine with Guanine to form base pairs. The base pairs, along with their respective sugar and phosphate molecules, form a Nucleotide. The Nucleotide forms a double helical structure, which looks like a ladder. Gene is the fundamental unit of heredity and is built-up of DNA. Genes are responsible for determining characteristics such as height, color, and many others. Some of the genes manufacture proteins, and some do not. According to the Human Genome Project, there are approximately around 25,000 genes in humans.

There are two copies of genes in every human; one passed on from the parent; almost all the genes are the same, except a few, less than 1% called the Alleles. They determine the unique physical features of a person. Genes manufacture proteins, and proteins, in turn, say what the cell should do (cell functions). The flow starts with DNA, RNA, and then the proteins. The flow of information determines the type of proteins being produced. The process in which the information contained in DNA is transformed into instructions to form proteins and other biochemical molecules is called gene expression. Gene expression assists the cells to react appropriately to the changing environment. The gene expression involves two critical steps in manufacturing the proteins, Transcription and Translation (Raut et al., 2010).

• Transcription: The DNA present in the gene will be copied to form an RNA known as the messenger RNA (mRNA). RNA is similar to DNA; however, it has a single-strand, and instead of Thymine, it has Uracil (U).
• Translation: The messages carried from the transcription by the mRNA will be read by the transfer RNA (tRNA) in the Translation phase. The mRNA can read three letters at a time, which constitutes one Amino acid (Amino acids are the building blocks of proteins).

Proteins play a significant role in cell functioning. Gene expression controls everything, such as when to produce protein, when not to, volume, i.e., increasing or decreasing the amount, etc. It is a kind of on/off switch. When this process does not happen as it is supposed to be, genetic disorders, tumors occur. A detailed study of the gene expression will help find the essential biomarkers that cause genetic disorders and tumors.

There are many techniques available to capture the gene expressions such as Northern blot, RNA protection assay, Reverse Transcription – Polymerase Chain Reaction (RT - PCR), Serial Analysis of Gene Expression (SAGE), Subtractive Hybridization, DNA Microarrays, Second Generation Sequencing (NGS) and many others. Among these, the most widely used these days is DNA Microarray (Raut et al., 2010; Wang and van der Laan, 2011). The DNA microarray technology manages to capture gene expressions of thousands of genes simultaneously. However, the Microarray result is enormous, with a high dimension, which makes the analysis challenging. Thus, it is necessary to perform gene selection to handle the high dimensional problem by removing the redundant and irrelevant genes. There are many computation techniques used in the field of bioinformatics been carried out over the years, such as Pattern Recognition, Data Mining, and many others to manage the high dimensional issue, yet ineffective (Raut et al., 2010).

Hence, in recent years, Machine Learning, which is a part of Artificial Intelligence, has gained the researchers’ attention in genomics and gene expression. Machine Learning is the part of Data Science; its primary purpose is to enable a model to train and learn to make decisions on its own in the future. Machine Learning is commonly categorized as Supervised, Unsupervised, and Semi-supervised or Semi-unsupervised learning. The Supervised involves the labeled data; unsupervised learning involves unlabeled data, and the Semi-supervised or Semi-unsupervised involves handling both labeled and unlabeled data. Machine Learning flows through Pre-processing and Classification or Clustering. In gene expression microarray data, machine learning-based feature selection approaches like gene selection approaches will help to select the required genes from the lot.

Feature selection helps in preserving the informative attributes. Feature selection is primarily applied to the high-dimensional data; in simple terms, feature selection is a dimensionality reduction technique (Kira and Rendell, 1992). Feature selection assists significantly in the fields, which have too many features and relatively scarce samples, for instance, RNA sequencing and DNA Microarray (Ang et al., 2015b).

The primary intent that feature selection got famous in the recent past is to extract the informative subset of features from the original feature space (Ang et al., 2015b). Feature selection techniques aids in overcoming the scare of model overfitting, handling the dimension, better interpretation of the feature space, maximizes prediction accuracy, and maximizes the model training time (Halperin et al., 2005; Sun et al., 2019b). The outcome of Feature selection is the optimal number of features that are relevant to the given class label, which contributes to the process of prediction.

One more technique for dimensionality reduction is Feature Extraction. Feature Selection is part of Feature Extraction (Cárdenas-Ovando et al., 2019). It is the process of transforming the original feature space into a prominent space, which can be a linear or non-linear combination of the original feature space (Anter and Ali, 2020). The major drawback of using Feature Extraction is that it alters the original feature space; eventually, the data interpretability is lost. Also, the transformation is usually expensive (Bermingham et al., 2015).

Gene expression is the flow of genetic information from Deoxy-ribose Nucleic Acid (DNA) to Ribose Nucleic Acid (RNA) to protein or other biomolecule syntheses. Gene expression data is a biological representation of various transcriptions and other chemicals found inside a cell at a given time. As data is recorded directly from DNA, through various experiments, a pertinent computational technique will reveal deep insights about the disease or disorder in the cell, eventually the organism in which the cell belongs (Koul and Manvi, 2020).

On the one hand, the gene expression data is highly dimensional; also, on the other, the sample size is incompetent. The high dimensionality in the data is due to the vast number of values generated for every gene in a genome in the order of thousands. Advanced technologies, for instance, Microarray, assists in analyzing thousands of proteins in a gene in a particular sample. However, the issue with Microarray is that it is expensive (Wahid et al., 2020).

However, the data with vast feature space will have redundant features with unnecessary information that will lead to overfitting, significantly affecting the model’s performance. The primary purpose of implementing the Feature selection or gene selection on gene expression data is to choose the most regulating genes and eliminate the redundant genes that do not contribute to the target class (Pearson et al., 2019).

The gene expression data are usually unlabeled, labeled, or semi-labeled, which leads to the necessity of the concepts of Unsupervised, Supervised, and Semi-supervised feature selection. Unlabeled data has no prior information about the functionalities, whereas it validates the gene selection based on data distribution, variance, and separability. Labeled data consists of meaningful class labels and information about the functionalities. Then gene selection will be performed based on the relevance and importance score of the labeled features. Semi-supervised or Semi-unsupervised combines a small amount of unlabeled data with labeled data and vice versa, which acts as additional information (Yang et al., 2019). This paper discusses the importance of feature selection or gene selection to have an improved result. This paper’s remaining sections discuss the background and development of feature selection, the steps involved in feature selection, a detailed discussion on various works on gene selection in the literature, the open issues, and future research directions concerning the gene expression data and conclusion.

The feature selection methods can be categorized into Supervised, Unsupervised, and Semi-supervised learning models. The survey works in the literature concentrate on either one of the models; for example (Kumar et al., 2017), focuses only on the supervised gene selection methods. Some works also concentrate on one particular feature selection strategy; for example (Lazar et al., 2012), focuses on filter-based techniques. Table 1 shows the comparison of existing reviews with the current survey. Our study categorizes the feature selection strategy into supervised, unsupervised, and semi-supervised methods and discusses the existing approaches in those categories. Also, we have done a detailed discussion of their performances.

**TABLE 1:** Comparison of existing reviews with the current survey.

References	Description	Shortcomings
Kumar et al., 2017	The survey focuses on the Supervised Gene Selection methods on Cancer Microarray dataset.	Concentrates only on Supervised Gene Selection methods.
Sheikhpour et al., 2017	The work discusses various works done in the Semi-Supervised Gene Selection methods, and the hierarchical structure of semi-supervised methods is also focused.	Concentrates only on Semi-Supervised Gene Selection methods.
Wang et al., 2016	The work focuses on the gene selection methods from a search strategy perspective.	Concentrates on search strategies in the feature selection methods.
Lazar et al., 2012	A survey on the filter-based feature selection techniques.	Concentrates on filter-based techniques in cancer microarray data.
Chandrashekar and Sahin, 2014; Mohamed et al., 2016	The work concentrates on various feature selection methods in microarray data.	In general, focus on the feature selection methods did not categorize as supervised, unsupervised, or semi-supervised.
Almugren and Alshamlan, 2019	A survey on the hybrid-based gene selection techniques.	Concentrates only on hybrid approach based gene selection methods.
**Current Survey**	Our survey on the existing literature focuses on the works mentioned above, categorizing into Supervised, Unsupervised, and Semi-Supervised Learning. Also, it discusses the performance of the existing gene selection methods.	

## Gene Selection – Background and Development

Gene Selection is the technique applied to the gene expression dataset, such as DNA Microarray, to reduce the number of genes, which are redundant and less expressive or less informative. Gene Selection has its base in the Machine Learning-based Feature Selection technique, which significantly suits the applications that involve thousands of features (Dashtban and Balafar, 2017). Gene Selection techniques are applied mainly for two reasons: finding the informative and expressive genes and removing the original space’s redundant genes. Theoretically, an increase in the number of genes will bring down the model’s performance and compromise the generalization by overfitting. The present works on Gene Selection concentrate mainly on finding the relevant genes, and there is limited research in removing the noise and redundant genes (Wang et al., 2005).

**FIGURE 1:**
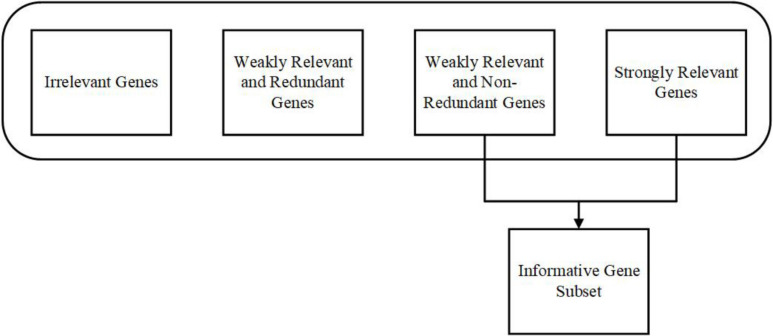
Representation of Gene Selection approach.

For significant results, it is critical to concentrate on relevancy, redundancy, and complementarity. A gene is considered as relevant when it has necessary information (individually or combined with other genes) about the given class, for example, tumorous or not. According to Yu and Liu (2004), the feature subset can be classified into strongly relevant, weakly relevant, and irrelevant in technical terms. The weakly irrelevant can again be classified into weakly relevant and redundant features and weakly relevant and non-redundant features. Most of the informative features can be found under strongly relevant and weakly relevant, and non-redundant features (Vergara and Estévez, 2014). The same approach is followed in the Gene Selection from the gene expression data. Figure 1 shows the representation of the Gene Selection approach.

Many works in literature (Hu et al., 2010; Hoque et al., 2014; Sun and Xu, 2014) aim to remove redundancy and relevancy from the data with the Mutual Information algorithm’s help in Gene Expression. Many variations in Mutual Information are implemented to tackle these two issues. Along with these two issues, there is one more issue, which many of the existing works fail to address, complementarity. Complementarity is the degree of feature interaction between a gene subset and an individual gene in a given class.

To solve the issues mentioned above, commonly, two approaches are followed in the literature, one is analyzing individual genes, and the other is finding an optimal subset. In analyzing individual genes, the genes are ranked based on their importance scores; genes with a similar score (redundant) and genes with the least score (irrelevant) below a given threshold will be removed. In finding an optimal subset, a search for a minimal subset of genes will be done, satisfying specific criteria and eliminating redundant and irrelevant genes.

In applications such as Text and Genomic Microarray analysis, the central issue is the “Curse of Dimensionality,” where finding the optimal subset of genes is considered an NP-hard problem. Effective learning will be achieved only when the model is trained with relevant and non-redundant genes. However, with an increase in the genes’ dimension, the possible number of optimal gene subsets will also increase exponentially.

In machine learning, feature space is defined as the space associated with a feature vector distributed all over the sample in an n-dimensional space. Moreover, to reduce the dimensionality of such feature space, feature extraction, or feature selection techniques can be used. Feature Selection is a part of the Feature Extraction technique. However, in feature selection, a subset from the original feature space will be formed, whereas, in feature extraction, a new set of feature space will be created that seems to capture the necessary information from the original feature space (Jović et al., 2015). The most commonly used feature extraction techniques are Principle Component Analysis (PCA), Independent Component Analysis (ICA), Expectation-Maximization (EM), and Linear Discriminant Analysis (LDA). Some examples of Feature Selection techniques are RELIEF, Conditional Mutual Information Maximization (CMIM), Correlation Coefficient, Information Gain, and Lasso (Khalid et al., 2014).

The major drawback of using Feature extraction is that the data’s interpretability will be lost in the transformation. Also, the transformation itself will be expensive sometimes (Khalid et al., 2014). Therefore, in this paper, we will discuss various Feature Selection techniques used in Gene Selection, which is less expensive and preserves the data’s interpretability.

The Gene Selection based on machine learning can be classified into three types, Supervised, Unsupervised, and Semi-Supervised. Supervised Gene Selection utilizes the genes that are labeled already (Filippone et al., 2006). The input and output labels are known in advance in this method. However, the data continues to grow and overwhelm the process, leading to data mislabeling, making it unreliable. The main issue in deploying Supervised Gene Selection is overfitting, which can be caused by selecting irrelevant or sometimes eliminating the most relevant gene (Ang et al., 2015b).

Unsupervised Gene Selection, unlike Supervised, will not have any labels to guide the selection process (Filippone et al., 2005). The data used in Unsupervised Gene Selection is unlabelled. That makes it unbiased and serves as an effective way to find the necessary insights into the classification process (Ye and Sakurai, 2017). The main issue in Unsupervised Gene Selection is that it does not consider the interaction among the Genes (correlation), making the resultant gene subset insignificant in the discrimination task (Acharya et al., 2017).

**FIGURE 2:**
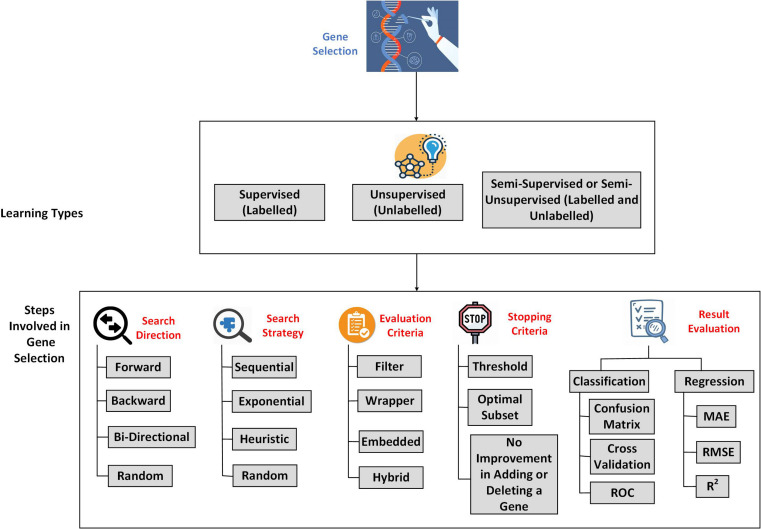
An overview of Gene Selection process.

Semi-supervised or Semi-unsupervised Gene Selection is like an add-on to the Supervised and Unsupervised Gene Selection. A Gene Selection is considered semi-supervised when most of the data is labeled, and a Gene Selection is said to be Semi-unsupervised when most of the data is unlabelled. The labeled data in the Semi-supervised or unsupervised is used to increase the distance between the data points that belongs to different classes, whereas the unlabelled data will help identify the geometrical structure of the feature space (Sheikhpour et al., 2017). Figure 2 illustrates the overview of the process involved in Gene Selection.

### Steps Involved in Feature Selection

#### Search Direction

The first stage involved in Feature Selection is to choose a search direction, which serves as a starting point to the process. There are three commonly used search directions:

• Forward Search: In Forward Search, the Search will be started with an empty set, and features are added one by one (Mohapatra et al., 2016).
• Backward Search: Search will be started with the whole set of genes, and the genes will be eliminated one by one with each iteration.
• Bi-directional: Search involves the advantages of Forward Search and Backward Search. The Search starts from both directions by either adding or removing a gene with each iteration (Abinash and Vasudevan, 2018). Other than these, Random Search is also used as a search direction (Wang et al., 2016).

#### Search Strategy

A good search strategy should attain fast convergence and provide an optimal solution with efficient computational cost and good global search ability (Halperin et al., 2005). There are three most widely used searching strategies:

• Sequential: follows a particular order in finding the best feature subset, for instance, Sequential Forward Search, where the search will be carried out from the start to the end (Chen and Yao, 2017). This strategy is prone to feature interaction and has the risk of attaining local minima (Wang et al., 2016). Examples: Floating Forward or Backward, Linear Forward Search, Beam Search, Greedy Forward Selection, and Backward Elimination.
• Exponential: It is a full-scale search; it guarantees an optimal solution but proves to be expensive. This approach finds all possible feature subsets to choose an optimal subset, which is computationally upscale, especially in high-dimensional datasets such as the Gene Expression Microarray dataset. Some of the examples for Exponential Search are, Exhaustive Search and Branch-and-bound.
• Heuristic Search: It is performed based on a cost measure or a heuristic function, which iteratively improves the solution. Heuristic Search does not always ensure an optimal solution, but it offers an acceptable solution with reasonable time, cost, and memory space (Ruiz et al., 2005). Some examples of Heuristic Search are Best-First Search, Depth-First Search, A^∗^ Search, Breadth-First Search, and Lowest-Cost-First Search (Russell and Norvig, 2016).

#### Evaluation Criteria

There are currently four types of evaluation methods used widely; they are Filter, Wrapper, Embedded, and Hybrid. Hybrid and Embedded methods are the recent developments in Gene Selection.

(a) Filter Feature Selection Approach:

Filter helps in identifying the specific abilities of features depending on the inherent properties of the data. The best among the features are identified with relevance score and threshold criteria (Hancer et al., 2018). The features with a low relevance score will be eliminated.

The significant advantages of filter techniques are that they are not dependent on the classifiers, fast and straightforward in terms of computation, and scaled to the immensely dimensioned dataset (Ang et al., 2015b). The common disadvantage is that they consider the data’s univariate features, which means the features are processed individually (Saeys et al., 2007). As a result, there are high chances of ignoring the feature dependencies, which leads to the classifiers’ poor performance compared to other feature selection approaches. Many multivariate filter techniques are introduced to avoid this to some extent (Brumpton and Ferreira, 2016; Djellali et al., 2017; Zhou et al., 2017; Rouhi and Nezamabadi-pour, 2018).

**FIGURE 3:**
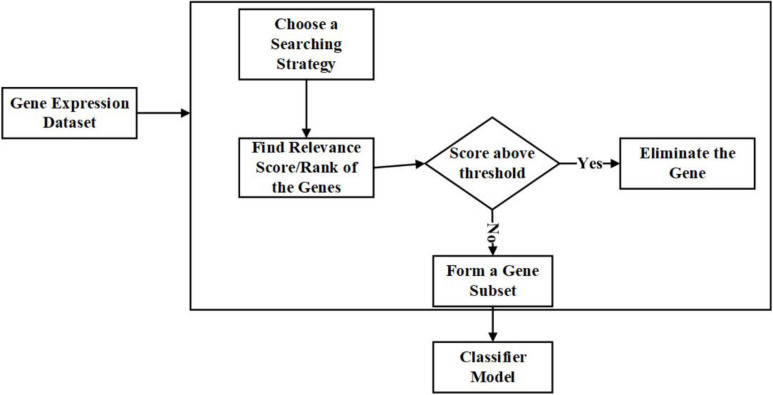
Flow diagram – Filter Feature Selection Approach.

The examples for filter techniques are Pearson Correlation, Fisher Score, Model-based Ranking, and Mutual Information (Lazar et al., 2012) were done in a detailed survey on the filter techniques applied to Gene Expression Microarray data. Figure 3 is the representation of the process involved in the filter approach in gene selection.

(b) Wrapper Feature Selection Approach:

Unlike the filter approaches, the wrapper approaches wrap the feature subset selection process around the black box’s induction algorithm. Once the search procedure for a feature subspace is defined, various feature subsets will be generated, and the classification algorithm is used to evaluate the selected feature subsets (Blanco et al., 2004). With this approach, it is possible to select features tailored for the induction algorithm (Jadhav et al., 2018). The classification algorithm’s evaluation measures will be optimized while eliminating the features, hence offering better accuracy than the filter approach (Inza et al., 2004; Mohamed et al., 2016).

**FIGURE 4:**
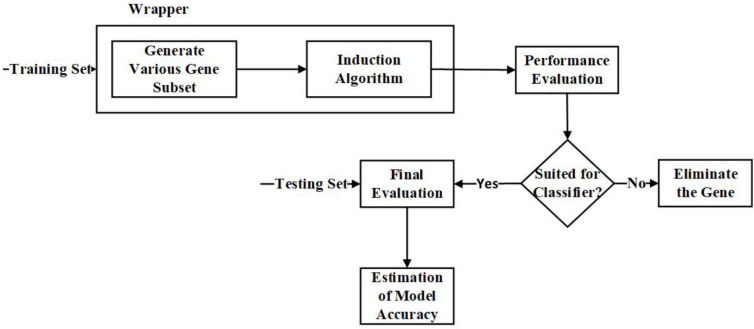
Flow diagram – Wrapper Feature Selection Approach.

The significant advantage of using a wrapper approach, as both feature subset generation and the induction algorithm are wrapped together; the model will have the ability to track the feature dependencies (Rodrigues et al., 2014). The common drawback is that it becomes computationally intensive for datasets with high dimensions (Mohamed et al., 2016). Examples of Wrapper techniques are Hill Climbing, Forward Selection, and Backward Elimination. Figure 4 is the representation of the process involved in the wrapper approach.

(c) Embedded Feature Selection Approach:

In a way, embedded approaches resemble the wrapper approaches, as both depend on the learning algorithm (Hernandez et al., 2007). However, the embedded methods are less computationally intensive than the wrapper methods. The link between the learning algorithm and the feature selection is more robust in embedded methods than the wrapper methods (Huerta et al., 2010). In the embedded methods, the feature selection is made as a part of the classification algorithm; in other terms, the algorithm will have its built-in approaches to select the essential features (Hira and Gillies, 2015).

**FIGURE 5:**
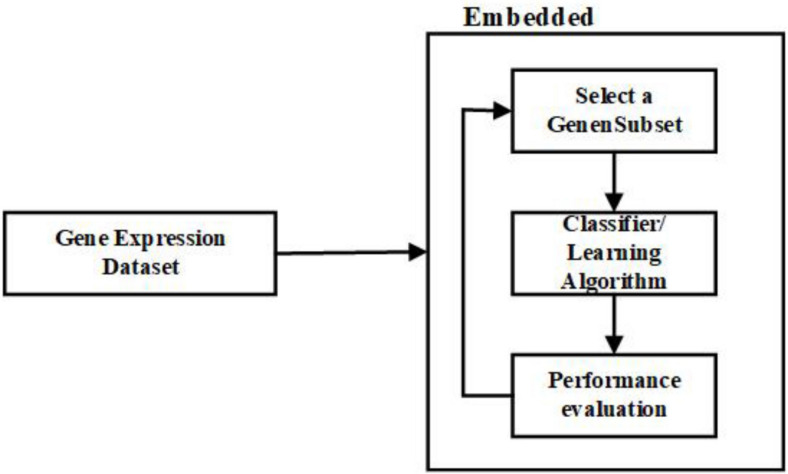
Flow diagram – Embedded Feature Selection Approach.

In the literature, it is mentioned that embedded methods combine the benefits of filter and wrapper methods to improve accuracy. The significant difference between other gene selection approaches and embedded approaches is how the genes are selected and the interaction with the learning algorithm (Chandrashekar and Sahin, 2014; Vanjimalar et al., 2018). Some examples of embedded approaches are ID3, RF, CART, LASSO, L1 Regression, and C4.5. Figure 5 is the representation of the process involved in the embedded approach.

**FIGURE 6:**
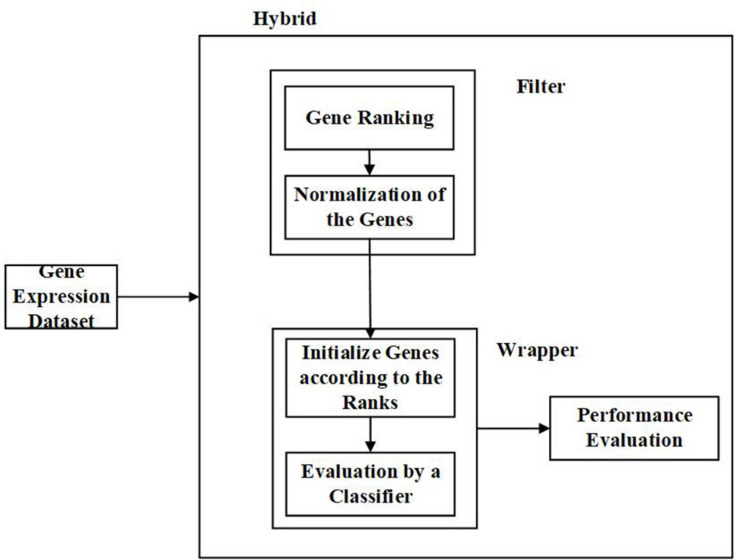
Flow diagram – Hybrid Feature Selection Approach.

(d) Hybrid Feature Selection Approach:

Hybrid methods, as the name suggests, is a combination of two different techniques. Here, it can be two different feature selection approaches or different methods with similar criterion or two different strategies. In most cases, the filter and wrapper approaches are combined to form a hybrid approach (Apolloni et al., 2016; Liu et al., 2019). It strives to utilize the benefits of two methods by combining their compatible strengths. Hybrid methods offer better accuracy and computational complexity than the filter and wrapper methods. Also, it is less susceptible to overfitting (Almugren and Alshamlan, 2019). Figure 6 is the representation of the process involved in the hybrid approach.

#### Stopping Criteria

The stopping criteria are a kind of threshold used to inform the classifier when to stop selecting the features (Wang et al., 2005). Appropriate stopping criteria will refrain a model from overfitting, thus offer better results, which are computationally cost-effective (Ang et al., 2015b). Some of the commonly used stopping criteria are as follows:

(1) When the search reaches a specific bound, the bound can be several iterations or many features.
(2) The results do not improve with a deletion (or addition) of another feature.
(3) An optimal subset is found. A subset is said to optimal when the classifier’s error rate is less than the preferred threshold.

#### Evaluating the Results

There are many performance evaluation metrics available in the literature to evaluate and validate the classifier results. In the classification case, i.e., predicting using the categorical attribute, the commonly used error estimation methods are Confusion Matrix, Cross-Validation, and Receiver Optimizer Characteristics (ROC). In the case of regression, i.e., predicting using the continuous attribute, the commonly used error estimation methods are Mean Absolute Error (MAE), Mean Squared Error (MSE), and Coefficient of Determination (R2).

(a) Confusion Matrix***:*** In the case of Multi-class problems, a confusion matrix is the best option to evaluate the classification model (Handelman et al., 2019). For instance, there are four possible results in a binary classification problem with which the model can be evaluated, True Positive, classified correctly, False Positive, erroneous classification, False Negative, erroneously rejected, and True Negative rejected correctly (Braga-Neto et al., 2004). Confusion Matrix offers measures such as Accuracy, Precision, Sensitivity, Specificity, and FMeasure to validate the results of a classifier.
(b) Cross-Validation (CV): It is the process of partitioning the available data into k-sets. Here, k can be any integer depending on the number of folds one needs for the classification or regression task (for instance, k = 10, k = 20, etc.) (Schaffer, 1993; Braga-Neto et al., 2004). CV is most commonly used on the Regression and Classification approaches (Chandrashekar and Sahin, 2014). The main advantage of using CV is that it offers unbiased error estimation, although sometimes it is variable (Bergmeir and Benítez, 2012).
(c) Receiver Optimization Characteristics (ROC): ROC graphs and curves are commonly used for visualizing the performance of the classifiers and select the one showing better performance (Landgrebe and Duin, 2008). As the researches these days are increasingly concentrated on the classification errors and unbalanced class distribution, ROC has gained a lot of attention (Flach, 2016). It is the depiction of the trade-offs between the Sensitivity or benefits (TPR) and the Specificity or costs (FPR) (Fawcett, 2006).
(d) Root Mean Square Error (RMSE): RMSE is a metric commonly used to measure the residuals’ standard deviation or prediction scores. In other words, the deviation in predictions from the regression line. It is given by Elavarasan et al. (2018),
  $$RMSE=\sqrt{\sum_{i=1}^{n}\frac{{\left(x_{i}-\bar{x_{i}}\right)}^{2}}{n}}$$
  Where, *x*_*i*_ – Actual or Observed Values.
  $\bar{x_{i}}$ – Predicted Values.
  *n* – Total number of sample.
(e) Mean Absolute Error: It is the standard measure of the residuals’ average magnitude (prediction errors), neglecting their directions. It is given by Elavarasan et al. (2018).
  $$MAE=\frac{1}{n}\sum_{i=1}^{n}\left|x_{i}-\bar{x_{i}}\right|$$
  Where, *x*_*i*_ – Actual or Observed Values,
  $\bar{x_{i}}$ – Predicted Values.
  *n* – Total number of sample.
(f) Determination Coefficient (R^2^): It is the measure to estimate how much one variable impacts other variables. It is the change in the percentage of one variable concerning the other. It is given by Elavarasan et al. (2018).
  $$R^{2}={\left[\frac{n\left[\sum \left(xy\right)-\left(\sum x\sum y\right)\right]}{\sqrt{n\sum x^{2}-{\left(\sum x\right)}^{2}\left[n\sum y^{2}-{\left(\sum y\right)}^{2}\right]}}\right]}^{2}$$
  Where, *x* – first set of values data,
  *y*– the second set of values in the data.
  *R* – Coefficient of determination.
  *n* – Total number of sample.

## Machine Learning Based Gene Selection Approaches

### Supervised Gene Selection

Supervised Gene Selection involves the data with labeled attributes. Most of the studies done in recent years have concentrated mainly on enhancing and improving the existing supervised gene selection methods.

For instance, Devi Arockia Vanitha et al. (2016) enhanced the Mutual Information (MI) filter method for selecting the informative gene. Also, Joe’s Normalized Mutual Information, an improved version of the standard existing MI approach, was implemented by Maldonado and López (2018). Filter approaches are independent of the classifiers used. Hence, many works are focused on developing filter technologies. For instance, a novel filter approach is mainly based on the Hilbert-Schmidt Independence Criterion (SHS) and motivate by Singular Value Decomposition (SVD). Table 2 shows some of the filter-based gene selection techniques used in the literature to select informative genes.

**TABLE 2:** Filter-based Supervised Gene Selection.

References	Ideology	Gene Selection Algorithm	Classifier	Dataset	Performance Evaluation Metrics
Ca and Mc, 2015	The informative genes are selected with the help and Mutual Information, which are then used to train the classifier.	Mutual Information	SVM (Linear, Quadratic, RBE and Polynomial), KNN, ANN	• Colon • Cancer • Lymphoma	• Error Rate • LOOCV
Shukla and Tripathi, 2019	The Spearman Correlation and Distributed Filters have been used to select the most significant genes.	Spearman Correlation and distributed filter	Naïve Bayes, Decision Tree, SVM, and kNN	• Breast Cancer • Colon Cancer • DLBCL • SBRCT • Prostate Cancer • Lung Cancer	• Accuracy • Precision • Sensitivity • FMeasure • ROC
Gangeh et al., 2017	The proposed method is based on the Hilbert Schmidt Independence Criterion, and it achieves scalability to large datasets and high computational speed.	Sparse Hilbert-Schmidt Independence Criterion (SHS)	SVM and kNN	• Lymphoma • Leukemia • Brain Tumor • 11_Tumors • SRBCT • Lung	• Classification Accuracy
Mazumder and Veilumuthu, 2019	In this study, a new method of Normalized Mutual Information called Joe’s Normalized Mutual Information (JNMI) had been developed and evaluated with five classifiers.	Joe’s Normalized Mutual Information	Naïve Bayes, Radical Function Network, Instance-based Classifier, Decision-based Table and Decision Tree	• Leukemia • Lymphoma • CNS • MLL • SRBCT	• Accuracy AUC

The wrapper approach is computationally intensive than other feature selection approaches. Works on the wrapper feature selection approach are less because of the issue mentioned above. So, most of the research on the wrapper is focused on improving the computational cost. For instance, Wang A. et al. (2017), Wang H. et al. (2017) implemented a wrapper-based gene selection with Markov Blanket, which reduces the computation time. Many approaches try to enhance the most widely used Support Vector Machine – Recursive Feature Elimination (SVM-RFE), such as Shukla et al. (2018), implemented Support Vector Machine – Bayesian t-test – Recursive Feature Elimination (SVM-BT-RFE), where Bayesian t-test is combined with SVM-RFE to improve the results. Table 3 shows the works done in recent years on Wrapper-based Supervised Gene Selection.

**TABLE 3:** Wrapper-based Supervised Gene Selection.

References	Ideology	Gene Selection Algorithm	Classifier	Dataset	Performance Evaluation Metrics
Wang A. et al., 2017	Aims to improve the evaluation time with the help of Markov Blanket with Sequential Forward Selection.	Wrapper-based Sequential Forward Selection with Markov Blanket	kNN, Naïve Bayes, C4.5 Decision Tree	• Colon • SRBCT • Leukemia • DLBCL • Prostate • Bladder • Gastric • Tox • Blastoma	• Classification Accuracy • Wilcoxon signed-rank test
Hasri et al., 2017	The proposed method Multiple Support Vector Machine – Recursive Feature Elimination is an enhancement of SVM-RFE for improving the accuracy in selecting the informative features.	MSVM-RFE	Random Forest, C4.5 Decision Tree	• Leukemia • Lung Cancer	• Classification Accuracy
Shanab et al., 2014	A wrapper-based feature selection technique has been developed with Naïve Bayes by using the real-world high dimensional data in terms of difficulty due to noise.	Naïve Bayes-Wrapper	Naïve Bayes, MLP, 5NN, SVM and Logistic Regression	• Ovarian • ALL AML Leukemia • CNS • Prostate MAT • Lymphoma • Lung Cancer	• AUC
Mishra and Mishra, 2015	This method aims to gather the relevant genes to distinguish the biological facts. The method is an extension of SVM-T-RFE, where instead of a t-test, a Bayesian t-test has been used for better results.	SVM- Bayesian T-Test –RFE (SVM-BT-RFE)	SVM-RFE, SVM-T-RFE	• Colon • Leukemia • Medulla Blastoma • Lymphoma • Prostate	• Classification Accuracy
Zhang et al., 2018	In this study, three wrapper based feature selections are implemented, and the results show that SVM-RFE-PSO performs better in selecting informative features than the other two.	SVM-RFE-GS, SVM-RFE-PSO, and SVM-RFE-GA	SVM	• Breast Cancer • TGCA	• AUC • Accuracy • Precision • Recall • F-Score

Hybrid Feature Selection is usually the combination of other approaches, mostly filter and wrapper approaches are made into hybrids. For instance, Liao et al. (2014), implemented a filter-wrapper based hybrid approach utilizing the Laplacian score and Sequential Forward and Backward Selection. Also, various works are going on in combining the nature-inspired algorithm. For example, Alshamlan et al. (2015), implemented a Genetic Bee Colony, combining the Genetic Algorithm and Artificial Bee Colony for gene selection. A hybrid of the Salp Swarm Algorithm (SSA) and multi-objective spotted hyena optimizer are implemented in Sharma and Rani (2019). The SSA focuses on diversity, and MOSHO concentrates on convergence. Table 4 consists of the recent works done on Hybrid-based Supervised Gene Selection approaches.

**TABLE 4:** Hybrid Supervised Gene Selection.

References	Ideology	Gene Selection Algorithm	Classifier	Dataset	Performance Evaluation Metrics
Zare et al., 2019	Addresses the linear independence to find informative features with the help of matrix factorization and SVD.	Matrix Factorization based on SVD	Naïve Bayes, C4.5, and SVM	• Brain • CNS • Colon • DLBCL • GLI • Ovarian • SMK • Breast • Prostrate	• Cross-Validation (5-Fold and DOB-SCV) • Sensitivity • Specificity • Accuracy • G-Mean
Chinnaswamy and Srinivasan, 2016	The correlation coefficient is used as the attribute evaluator and PSO as a search strategy to select the necessary features.	Correlation Coefficient and PSO	ELM, J48, Random Forest, Random Tree, Decision Stump, and Genetic Programming	• SRBCT • Lymphoma • MLL	• Classifier Accuracy
Alshamlan et al., 2015	The Genetic Bee Colony combines the benefits of the Genetic Algorithm and Artificial Bee Colony. The method is evaluated using SVM.	Genetic Bee Colony	SVM	• Colon • Leukemia • Lung • SRBCT • Lymphoma	• Classification Accuracy • LOOCV
Liao et al., 2014	Two-stage feature selection methods involve the Laplacian Score and wrapper approach (SFS and SBS) to select the superior genes. Also, it considers the variance information.	Locality Sensitive Laplacian Score, Sequential Forward Selection and Sequential Backward Selection	SVM	• Acute Lymphoma • Lung Cancer • DLBCL • Prostrate • MLL Leukemia • SRBCT	• Accuracy • Precision • Recall • F-Score • AUROC
Shukla et al., 2018	This hybrid method targets at improving the classification accuracy with a two-stage method. It comprises the EGS (multi-layer and F-Score approach) as the first stage to reduce the noise and redundant features; in the second stage, AGA is used as a wrapper to select the informative genes used SVM and NB as fitness functions.	Multi-Layer Ensemble Gene Selection (EGS) and Adaptive Genetic Algorithm (AGA)	SVM and Naïve Bayes	• Breast • Colon • DLBCL • SBRCT • Lung • Leukemia	• Accuracy • FMeasure • Sensitivity
Sun et al., 2019a	A hybrid gene selection method combining the ReliefF and the Ant Colony Optimization is proposed. It is a filter-wrapper based gene selection.	ReliefF-Ant Colony Optimization	RFACO-GS	• Colon • Leukemia • Lung • Prostrate	• Classification Accuracy

Ensemble Feature Selection is a combination of the outputs from different expert feature selection approaches. Ghosh et al. (2019a; 2019b), combines the outputs of ReliefF, Chi-square, and Symmetrical Uncertainty (SU) with Union and Intersection of top “n” features. Seijo-Pardo et al. (2016), used a ranking aggregation method to various aggregate ranks from Chi-square, InfoGain, mRmR, and ReliefF. Table 5 shows the different Ensemble-based Supervised Gene Selection approaches used in recent years.

**TABLE 5:** Ensemble-based Supervised Gene Selection.

References	Ideology	Gene Selection Algorithm	Classifier	Dataset	Performance Evaluation Metrics
Ghosh et al., 2019a	The three filter methods are made into an ensemble with the Union and Intersection of top n features, which are then further fine-tuned using the Genetic Algorithm.	Relief F, Chi-Square, and Symmetrical Uncertainty.	KNN, MLP, and SVM	• Colon • Lung • Leukemia • SRBCT • Prostrate	• Accuracy
Seijo-Pardo et al., 2016	The proposed method combines different individual rankings with various aggregation methods. The methods used are Chi-Square, InfoGain, mRMR, and ReliefF.	Ranker Ensemble	SVM-RBF Kernel	• Colon • DBCL • CNS • Leukemia • Lung • Prostate • Ovarian	• Error Rate
Xu et al., 2014	The Correlation based feature selection incorporating the Neighborhood Mutual Information (NMI) and Particle Swarm Optimization (PSO) are combined into an ensemble (NMICFS-PSO) for cancer recognition.	NMICFS – PSO	SVM	• Breast • DLBCL • Leukemia • Lung • SRBCT	• LOOCV • Classification Accuracy
Yang et al., 2016	The authors have designed an ensemble based feature selection for a multi-class classification problem. The study aims to show that balanced sampling and feature selection together assists in improving the results.	Iterative Ensemble Feature Selection (IEFS)	SVM and kNN	• GLM • Lung • ALL • ALL-AML-4 • ALL-AML-3 • Thyroid	• AUC
Brahim and Limam, 2018	A robust aggregator technique has been proposed by combining the reliability assessment and classification performance based on the expert algorithms’ outputs.	Reliability Assessment-based Aggregation	kNN	• DLBCL • Bladder • Lymphoma • Prostate • Breast • CNS • Lung	• k-Fold Cross-Validation (k = 10)
Boucheham et al., 2015	A two-staged wrapper-based ensemble gene selection method has been implemented to identify the gene expression data’s biomarkers. A filter-based approach and parallel metaheuristics were performed at every stage in the ensemble.	Ensemble of Co-operative Parallel Metaheuristics	-	• 9_tumors • 11_tumors • Prostate • Colon • Leukemia • Ovarian • DLBCL • SRBCT • Brain Tumor	• Accuracy • Jaccard Index • Kuncheva Index

Embedded methods merge the benefits of filter and wrapper methods, where the learning algorithm has a built-in feature selection approach. Ghosh et al. (2019b), implemented a Recursive Memetic Algorithm (RMA) with a wrapper-based approach embedded in it. Also, Guo et al. (2017), used L1 Regularization, along with a feature extraction method for selecting the informative genes. Table 6 shows the various Embedded-based Supervised Gene Selection approaches developed in recent years.

**TABLE 6:** Embedded-based Supervised Gene Selection.

References	Ideology	Feature Selection Algorithm	Classifiers	Datasets	Performance Evaluation Metrics
	The IDGA uses Laplacian and Fisher score as ranking measures and a genetic algorithm to select the informative features.	Intelligent Dynamic Genetic Algorithm (IDGA)	KNN, SVM, Naïve Bayes	• SRBCT • Breast • DLBCL • Leukemia • Prostrate	• LOOCV
Ghosh et al., 2019b	The wrapper approach is embedded in the RMA algorithm to find informative features.	Wrapper based Recursive Memetic Algorithm	SVM, MLP, and KNNS	• AMLGSE2191 • Colon • Leukemia • MLL • SRBCT • Prostrate	• Accuracy • 5-Fold Cross-Validation • LOOCV
Guo et al., 2017	This study’s embedded method is a two-stage method with feature selection and feature extraction, L1 regularization as the feature selection method, and Partial Least Square (PLS) as the feature extraction.	L1 Regularization	LDA	• GCM • MLL • GLIOMA • Lung • SRBCT • NCI60 • Breast • CLL-SUB-111 • GLA-BAR-180 • DLBCL	• Classification Accuracy • CPU Time • Sensitivity
Wang H. et al., 2017	This method targets minimizing the computational cost and maximizing the performance by selecting a minimal number of necessary genes. This method distinguishes the features by their occurrence frequency and classification performance.	Weighted Bacterial Colony Optimization	Sequential Minimal Optimization (SMO) and kNN	• Breast Cancer Wisconsin • CNS • Colon • Leukemia • 9_Tumors • 11_Tumors • Brain • SRBCT • Prostate • DLBCL	• Classification Error Rate • Classification Accuracy
Maldonado and López, 2018	With the scaling factors approach’s help, the embedded strategy proposed in this study penalizes the feature cardinalities.	Kernel Penalized-Support Vector Data Description (KP-SVDD) and Kernel Penalized-Cost Sensitive Support Vector Machine (KP-CSSVM)	SVM	• GORDAN • GLIOMA • SRBCT • BHAT • CAR • BULL	• Classification Accuracy
Algamal and Lee, 2015	The embedded approach proposed implements the adaptive LASSO, which focuses on solving the initial weight uncertainty issue	Adaptive LASSO (APLR)		• Colon • Prostrate • DLBCL	• AUC • Misclassification Error

### Unsupervised Gene Selection

Unsupervised Gene Selection involves data without any labels. Compared to Supervised Gene Selection, works on Unsupervised are less.

There are many novel works done on filter-based unsupervised gene selection, such as Solorio-Fernández et al. (2017), proposed a filter method for both non-numerical and numerical data. It is a combination of kernel approach and spectrum-based feature evaluation. Also, Liu et al. (2018), developed a Deep Sparse Filtering model considering the deep structures, enhancing the results. Many studies on nature-inspired gene selection and the (Guo et al., 2017) implemented the MGSACO to minimize redundancy, thereby increasing the dataset**’**s relevancy. One another issue with high-dimensional data is dependency maximization. The work in Boucheham et al. (2015) implemented the Hilbert-Schmidt Independence Criterion to eliminate the most dependent genes to handle dependency maximization. Table 7 is the collection of works done in recent years on Filter-based Unsupervised Gene Selection approaches.

**TABLE 7:** Filter-based Unsupervised Gene Selection.

References	Ideology	Gene Selection Algorithm	Classifier	Dataset	Performance Evaluation Metrics
Solorio-Fernández et al., 2017	A new filter based unsupervised gene selection method, which can be used for numerical and non-numerical data, has been proposed. It is a combination of a spectrum based feature evaluation and a kernel.	Unsupervised Spectral Feature Selection Method (USFSM)	SVM, kNN, and Naïve Bayes	• Heart • Liver • Dermatology Thoracic	• AUROC • Accuracy K-Fold (k = 5)
Liaghat and Mansoori, 2016	HSIC is a framework for unsupervised gene selection that considers the dependency maximization among the similarity matrices after eliminating a gene.	Hilbert-Schmidt Independence Criterion (HSIC)	Gap-Statistics and k-Means	• Several microarray datasets	• Accuracy
Tabakhi et al., 2015	The Ant Colony Optimization is used as a Filter approach to maximize the relevance scores among the genes and minimize the redundancy.	Microarray Gene Selection based on Ant Colony Optimization (MGSACO)	SVM, Naïve Bayes, and Decision Tree	• Colon • Leukemia • SRBCT • Prostate • Lung Cancer	• Classification Error Rate
Liu et al., 2018	An unsupervised gene selection by implementing the sparse filtering and sample learning as a filter approach was proposed. It takes into consideration deep structures, which helps in obtaining improved results.	Sample Learning based on Deep Sparse Filtering (SLDSF)	-	• DLBCL • Lung Cancer • Leukemia • Esophageal Cancer (ESCA) • Squamous cell Carcinoma Head and Neck (HNSC)	• *p*-Values of GO terms

Filter-based gene selection approaches are not dependent on the learning model; on the contrary, wrapper methods are entirely dependent on the learning model. The dependency makes it complicated and has a high computational cost. Hence, the study on wrapper methods is less concentrated. Same with the unsupervised wrapper gene selection, which is less focused. Xu et al. (2017), has implemented SVM-RFE, a wrapper-based gene selection, on unlabeled data to distinguish high-risk and low-risk cancer patients. Table 8 is an example of a wrapper-based Unsupervised Gene Selection approach.

**TABLE 8:** Wrapper-based Unsupervised Gene Selection.

Reference	Ideology	Gene Selection Algorithm	Classifier	Dataset	Performance Evaluation Metrics
Xu et al., 2017	In this study, a standard Support Vector Machine – Recursive Feature Elimination was performed on microarray data to distinguish low-risk and high-risk colon cancer patients.	SVM-RFE	SVM	• GSE38832 • GSE17538 • GSE28814 • TGCA	• AUROC • Accuracy • K-Fold (k = 5)

Hybrid Unsupervised gene selection is also focused on in the literature as much as the filter approach. Li and Wang (2016), developed a two-stage gene selection approach; it applies the matrix factorization and minimum loss principle. A coarse-fine hybrid gene selection on unlabelled data shows better results than a few other approaches compared to the study. Filter-wrapper hybrid approaches are equally focused on supervised as well as unsupervised gene selection. For instance, Solorio-Fernández et al. (2017), implemented a Laplacian Score Ranking, a filter approach, and Normalised Calinski-Harabasz (LS-WNCH), a wrapper approach as hybrid unsupervised gene selection. It includes the properties of spectral feature selection. Table 9 shows the hybrid-based Unsupervised Gene Selection approaches.

**TABLE 9:** Hybrid Unsupervised Gene Selection.

References	Ideology	Gene Selection Algorithms	Classifier	Dataset	Performance Evaluation Metrics
Solorio-Fernández et al., 2016	A filter-wrapper based hybrid gene selection method having the properties of spectral feature selection, Laplacian Score Ranking, and enhanced Calinski-Harabasz Index.	Laplacian Score Ranking – Weighted Normalized Calinski Harabasz (LS – WNCH)	k-Means	• Lymphoma • Tumors • Leukemia	• Jaccard Index
Manbari et al., 2019	To solve the issue of high-dimension and the search space, a filter-wrapper based hybrid gene selection has been proposed with a clustering and improved Binary Ant System.	Feature Selection based on Binary Ant System (FSCBASM)	SVM, kNN, and Naïve Bayes	• Colon • Leukemia	• Accuracy • FMeasure • Recall • Precision

Ensemble and embedded approaches are studied less than the filter and hybrid methods. Elghazel and Aussem (2013), implemented a Random Cluster Ensemble with k-means as the clustering model. The ECE was constructed with different bootstrap samples at every ensemble partitions. They have also calculated out-of-bag feature importance at every ensemble. Li et al. (2017), developed a Reconstruction-based unsupervised feature selection model, an embedded approach. The model has a filter-based approach embedded in the k-means clustering. Table 10 is the example for Ensemble-based, and Embedded-based Unsupervised Gene Selection approaches.

**TABLE 10:** Ensemble and embedded Unsupervised Gene Selection.

References	Ideology	Gene Selection Algorithms	Classifier	Dataset	Performance Evaluation Metrics
Li et al., 2017	A reconstruction based gene selection has been proposed to perform a data independent filter-based gene selection embedded in the approach. (Embedded)	Reconstruction-based Unsupervised Feature Selection (REFS)	k-Means	• Lung • GLIOMA	• Accuracy • Normalized Mutual Information
Elghazel and Aussem, 2015	The RCE was constructed with a random set of features different bootstrap samples at each partition. The out-of-bag feature importance was calculated from every ensemble partition. (Ensemble)	Random Cluster Ensemble (RCE)	k-Means	• Leukemia • Ovarian Lung	• Accuracy • Normalized Mutual Information (NMI)

### Semi-Supervised Gene Selection

Semi-supervised gene selection is yet to be explored research area. There are not many works done as much as supervised or unsupervised gene selection. Semi-Supervised or Semi-Unsupervised consists of both labeled and unlabelled data.

Li et al. (2018), combined the benefits of the spectral graph and Mutual Information to develop a Semi-Supervised Maximum Discriminative Local Margin (SemiMM). It takes care of variance, local structure, and MI all at the same time. SVM is used widely in supervised and unsupervised gene selection approaches; in semi-supervised, Ang et al. (2015b), implemented a semi-supervised SVM-RFE (S3VM) for selecting the informative genes, and it proves to be successful. Chakraborty and Maulik (2014), developed a hybrid model; Kernalised Fuzzy Rough Set (KFRS) and S3VM are combined to select the relevant features. The results show that the proposed algorithm is capable of choosing useful biomarkers from the dataset. A semi-supervised embedded approach, Joint Semi-Supervised Feature Selection (JSFS), was developed with a Bayesian approach. The model automatically chooses the informative features and also trains the classifier.

Rajeswari and Gunasekaran (2015), developed an ensemble-based semi-supervised gene selection to improve the quality of the cluster model. Modified Double Selection based Semi-Supervised Cluster Ensemble (MDSVM-SSCE) assists in selecting the most relevant genes. Table 11 shows the Semi-Supervised Gene Selection approaches developed in recent years.

**TABLE 11:** Semi-Supervised Gene Selection approaches.

References	Ideology	Gene Selection Algorithm	Classifier	Dataset	Performance Evaluation Metrics
Ang et al., 2015a	An SVM-based semi-supervised gene selection technique has been proposed. The results show better performance in terms of accuracy and process time than other standard Supervised gene selection techniques. (Wrapper)	Semi-Supervised SVM-based RFE (S^3^VM-RFE)	SVM	• Lung Cancer	• K-fold Cross-Validation • (k = 10)
Li et al., 2018	A filter-based feature selection called SemiMM was proposed, which handles mutual information, local structure, and variance at the same time. It is a combination of mutual information and spectral graph. (Filter)	Semi-Supervised Maximum Discriminative Local Margin (SemiMM)	SVM	• DLBCL • Prostate • Tumor • Leukemia2 • SRBCT • Lung Cancer	• Accuracy • Precision • Recall • FMeasure • AUC
Rajeswari and Gunasekaran, 2015	The authors have proposed an ensemble-based framework aiming to improve the quality of the clustering model. The double selection cluster ensemble feature selection assists in selecting the most relevant genes. (Ensemble)	Modified Double Selection-based Semi-Supervised Cluster Ensemble (MDSVM-SSCE)	PC-K-means Clustering approach.	• Tumors	• Normalized Mutual Information (NMI)
Chakraborty and Maulik, 2014	The SVM model has been combined with Fuzzy Rough Set as a Semi-Supervised approach to select the informative features. The proposed algorithm proves to be capable of selecting useful biomarkers from the datasets. (Hybrid)	Kernalised Fuzzy Rough Set (KFRS) S^3^VM	Transductive SVM (TSVM)	• SRBCT • DLBCL • Leukemia • MicroRNA	• T-Statistics • Wilcoxon Signed-Rank test • AUC • FMeasure
Jiang et al., 2019	An embedded method, with the Bayesian approach. It automatically chooses the informative features and also trains the classifier. (Embedded)	Joint Semi-Supervised Feature Selection and Algorithm (JSFS)	Bayesian approach to select and classify	• Prostate • Colon	• Accuracy
Liang et al., 2016	The most widely adopted for high and low-risk classification. The L ½ regularization has been embedded in these models to select appropriate and relevant genes to enhance the models’ performance. (Embedded)	L ½ Regularization	CoX and AFT Models	• Tumor	• Precision

## Performance Analysis and Discussion on the Reviewed Literature

In the literature, the top three datasets used widely are Prostate, Leukemia, and Colon. Tables 12–14 shows the respective proposed models’ performance on the datasets mentioned above, along with the number of genes selected.

**TABLE 12:** Performance analysis of prostate dataset.

Category	Literature	Performance Analysis	Type pf Metric Used	Selected No. of Genes
**Supervised Feature Selection**	Shukla and Tripathi, 2019	99.81%	Accuracy	-
	Wang A. et al., 2017	88.3%	Accuracy	12
	Mishra and Mishra, 2015	99.64%	Accuracy	20
	Zare et al., 2019	92%	Accuracy	25
	Liao et al., 2014	86.76%	Accuracy	52
	Ghosh et al., 2019a	98.03%	Accuracy	7
	Seijo-Pardo et al., 2016	2.94	Error Rate	89
	Brahim and Limam, 2018	82%	10-Fold CV	20
		100%	LOOCV	18
	Ghosh et al., 2019b	95.1%	5-Fold CV	5
**Unsupervised Gene Selection**	Tabakhi et al., 2015	26.85	Error Rate	20
**Semi-Supervised Gene Selection**	Li et al., 2018	90%	Accuracy	150
	Jiang et al., 2019	91%	Accuracy	30

**TABLE 13:** Performance analysis on Leukemia dataset.

Category	Literature	Performance Analysis	Type pf Metric Used	Selected No. of Genes
**Supervised Feature Selection**	Gangeh et al., 2017	98.61%	Accuracy	1000
	Wang A. et al., 2017	95.5%	Accuracy	4
	Alshamlan et al., 2015	100%	LOOCV	200
	Liao et al., 2014	97.79%	Accuracy	-
	Shukla et al., 2018	94.34%	Accuracy	13
	Ghosh et al., 2019a	100%	Accuracy	12
	Seijo-Pardo et al., 2016	14.71	Error Rate	5
	Xu et al., 2014	99%	10-Fold CV	15
	Boucheham et al., 2015	100%	Accuracy	25
		94.1%	Accuracy	15
	Ghosh et al., 2019b	96.1%	5-Fold CV	5
**Unsupervised Gene Selection**	Tabakhi et al., 2015	23.07	Error Rate	20
	Manbari et al., 2019	94.8%	FMeasure	40
	Jain et al., 2018	97.2%	Accuracy	3
**Semi-Supervised Gene Selection**	Elghazel and Aussem, 2015	95%	Accuracy	150
	Chakraborty and Maulik, 2014	98%	Accuracy	20

**TABLE 14:** Performance analysis of colon dataset.

Category	Literature	Performance Analysis	Type pf Metric Used	Selected No. of Genes
**Supervised Feature Selection**	Ca and Mc, 2015	11	Error Rate	200
	Shukla and Tripathi, 2019	99.1%	Accuracy	13
	Wang A. et al., 2017	82.9%	Accuracy	1000
	Mishra and Mishra, 2015	99.5%	Accuracy	25
	Zare et al., 2019	93%	Accuracy	15
	Alshamlan et al., 2015	96.7%	Accuracy	5
	Shukla et al., 2018	83.54%	Accuracy	5
	Ghosh et al., 2019a	100%	Accuracy	-
	Seijo-Pardo et al., 2016	20	Error Rate	15
	Ghosh et al., 2019b	100%	5-Fold CV	12
	Wang H. et al., 2017	98.25%	Accuracy	4
**Unsupervised Gene Selection**	Tabakhi et al., 2015	23.63	Error Rate	20
	Manbari et al., 2019	95%	Accuracy	40
**Semi-Supervised Gene Selection**	Jiang et al., 2019	87%	Accuracy	120
				

**FIGURE 7:**
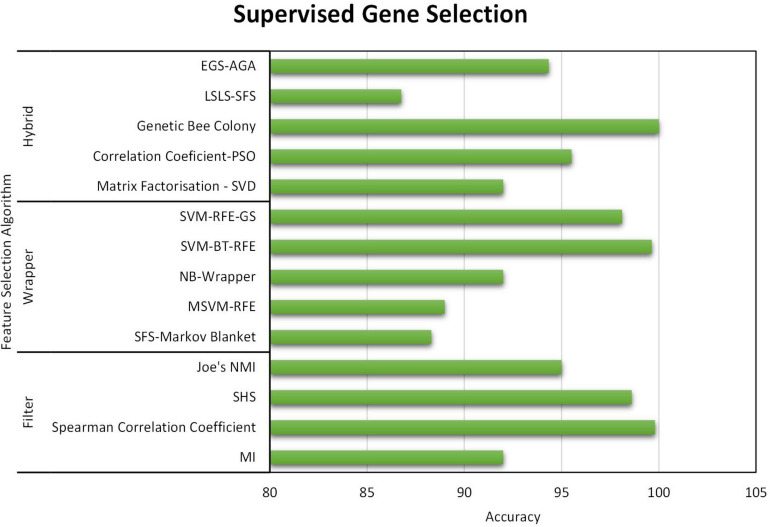
Performance analysis of Supervised Gene Selection Models – Part A.

**FIGURE 8:**
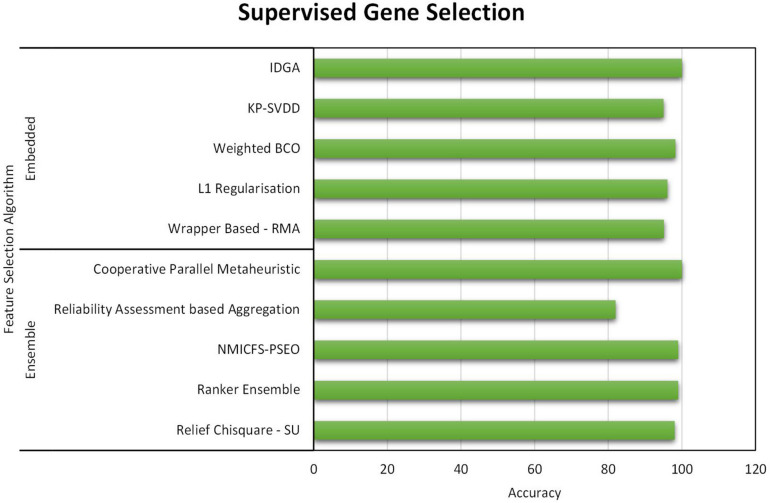
Performance analysis of Supervised Gene Selection Models – Part B.

**FIGURE 9:**
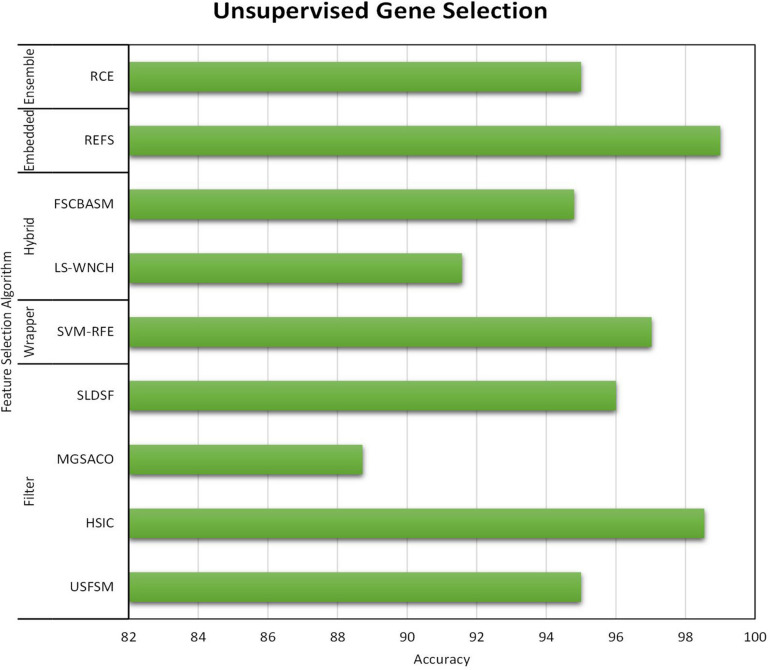
Performance analysis of Unsupervised Gene Selection Models.

**FIGURE 10:**
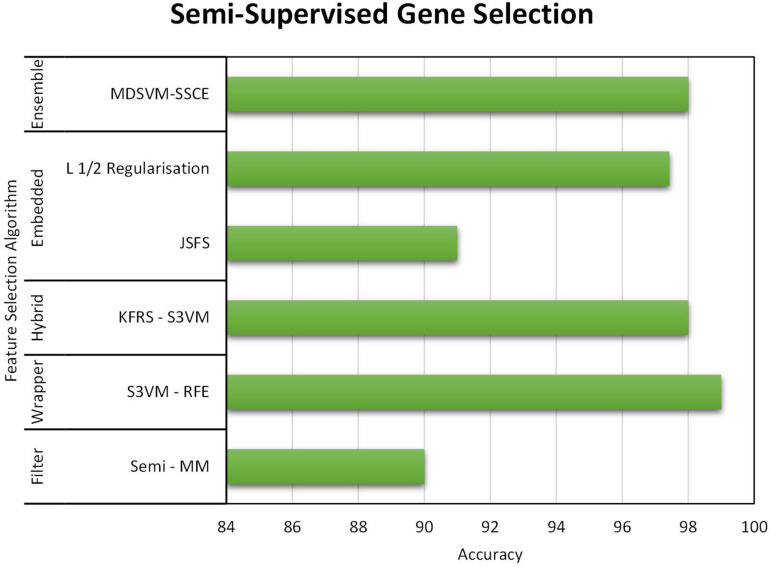
Performance analysis of Semi-Supervised Gene Selection Models.

All three gene selection methods discussed in this paper has its own merits and demerits. From the literature, it is clear that the Supervised Gene Selection is researched the most in recent years, and the Semi-supervised the least. Even though the Semi-Supervised potential is not tapped upon yet, it seems to be the better one among the three. It takes the advantages of Supervised and Unsupervised Gene Selection approaches. It has both labeled and unlabelled data; thus, it combines both the approaches’ benefits, eventually achieving better results. It considers the overlapping genes and handles it with the Unsupervised Gene Selection approach (unlabelled data) and learn and train the learning model with great accuracy and precision with the help of Supervised Gene Selection approaches (labeled data). Figures 7–10 show that the Supervised Gene Selection performs way better than the other two. Still, it might be because there are considerably significantly fewer works in Unsupervised and Semi-Supervised Gene Selection. The abbreviations for the acronyms used in the plot can be found in Table 15. There are several opportunities still untapped in these two areas. We can also notice that many works are concentrated more on Filter approaches as they are simple and computationally effective. However, hybrid approaches are upcoming and promising.

**TABLE 15:** Acronyms.

Acronyms	
EGS-AGA	Multi-Layer Ensemble Gene Selection (EGS) and Adaptive Genetic Algorithm (AGA)
LSLS-SFS	Locality Sensitive Laplacian Score, Sequential Forward Selection
PSO	Particle Swarm Optimization
SVD	Singular Vector Decomposition
SVM-RFE-GS	Support Vector Machine-Recursive Feature Elimination-Grid Search
SVM-BT-RFE	Support Vector Machine-Bayesian T test-Recursive Feature Elimination
NB	Naïve Bayes
MSVM-RFE	Multiple Support Vector Machine-Recursive Feature Elimination
SFS-MB	Sequential Forward Selection-Markov Blanket
NMI	Normalized Mutual Information
IDGA	Intelligent Dynamic Genetic Algorithm
KP-SVDD	Kernel Penalized-Support Vector Data Description
BCO	Bee Colony Optimization
RMA	Recursive Memetic Algorithm
RCE	Random Cluster Ensemble
REFS	Reconstruction-based Unsupervised Feature Selection
FSCBASM	Feature Selection based on Binary Ant System
LS-WNCH	Laplacian Score – Weighted Normalized Calinski-Harabasz
SLDSF	Sample Learning based on Deep Sparse Filtering
MGSACO	Microarray Gene Selection based on Ant Colony Optimization
HSIC	Hilbert-Schmidt Independence Criterion
USFSM	Unsupervised Spectral Feature Selection Method
MDSVM-SSCE	Modified Double Selection based Semi-Supervised Cluster Ensemble
JSFS	Joint Semi-Supervised Feature Selection
KFRS-S3VM	Kernalised Fuzzy Rough Set
Semi-MM	Semi-Supervised Maximum Discriminative Local Margin

As for the evaluation criteria, in recent years, filter-based approaches are more focused much. Filter methods function independently of the learning model; thus, it is less computationally intensive. As it is less complicated, many researchers target the filter-based approaches in selecting informative genes. Wrapper-based approaches are the least concentrated upon; it is dependent and designed to support the learning model. Wrapper approaches are usually time-consuming and generate high computational overhead. Though other methods are concentrated equally, the hybrid approach proves to be better among the others. Hybrid is a combination of two or more approaches. The most commonly used hybrid method is the Filter-Wrapper combination. In the Hybrid approach, the limitations of the individual approaches are compensated; in other words, it inherits the benefits of two methods. Further, this will minimize computational cost. Hybrid approaches seem to provide better accuracy and reduce over-fitting risks. Apparently, hybrid methods are most suited for high-dimensional datasets such as the gene expression microarray from the literature.

Apart from the discussed literature, many other works focused on nature-inspired and meta-heuristic algorithms in diagnosing cancer. A bio-inspired algorithm is proposed by Dashtban et al. (2018) using the BAT algorithm with more refined and effective multi-objectives. Also, they have proposed a novel local search strategy. Another such BAT inspired algorithm with two-staged gene selection is proposed in Alomari et al. (2017), wherein the first stage is a filter (Minimum Redundancy and Maximum Relevance) and the second stage is the wrapper consisting of BAT and SVM. Other than that, considerable works are done in Particle Swarm Optimization (PSO) by improving and enhancing the existing algorithm. In Jain et al. (2018), the authors implemented a two-phased hybrid gene selection method, combining the improved PSO (iPSO) and Correlation-based Feature Selection (CFS). The proposed method controls the early convergence problem. A recursive PSO is implemented in Prasad et al. (2018); it tries to refine the feature space into more fine-grained. They have also combined existing filter-based feature selection methods with the recursive PSO. KNN and PSO are implemented in Kar et al. (2015) to handle the uncertainty involved in choosing the k-value in KNN. In Han et al. (2015), the authors proposed a Binary PSO (BPSO) to improve the interpretability of the gene selected and improve the prediction accuracy of the model. In Shreem et al. (2014), a nature-inspired algorithm Harmony Search Algorithm (HAS) is embedded with Markov Blanket, which focuses on symmetrical uncertainty Sharbaf et al. (2016) implemented an Ant Colony Optimization based gene selection (ACO) along with Cellular Learning Automata (CLA) as a wrapper method. In another approach (Lai et al., 2016), a hybrid combining filter and wrapper approaches is implemented using Information Gain (IG) and improved Swarm Optimization to find the optimal gene subset. Information Gain (IG) is also implemented along with SVM in Gao et al. (2017) to remove the redundant genes. There are works done in gene selection using the Genetic algorithms with different variations from the existing one. One such work combines the Genetic algorithm and Fuzzy in Nguyen et al. (2015), integrating the two approaches to finding out the optimal gene subset. Genetic Algorithm is also combined with learning automata (GALA) in Motieghader et al. (2017), which improves the time complexity in selecting the gene subset. Statistically, significant models are also implemented, such as the entropy-based measure and rough sets (Chen et al., 2017) and (Xiao et al., 2014; Sun et al., 2019c), testing the statistical significance with p-value and fold change. Decision tree and random forest variances are also worked on, such as the four-state-of art Random forest (Kursa, 2014), decision tree along with PSO (Chen et al., 2014), and a guided regularized Random Forest (Deng and Runger, 2013). Various works are focus on improving the interpretability of the features and reducing the feature space with improvements in the existing models (Zibakhsh and Abadeh, 2013; Cai et al., 2014; García and Sánchez, 2015; Chen et al., 2016; Tang et al., 2018; Cleofas-Sánchez et al., 2019).

Machine Learning techniques are widely used in modern-day research in the field of bioinformatics. The Machine Learning algorithms are available under different criteria, such as the logic-based algorithms (E.g., Decision Trees, Random Forest), perceptron-based algorithms (Neural Network, Multi-layered Perceptron), and Statistical Learning (Naïve Bayes) (Kotsiantis et al., 2007). The classification or prediction models used commonly in the literature discussed in this paper mostly include SVM, KNN, Random Forest, Decision Tree, Naïve Bayes, and Logistic Regression. SVM consists of support vectors that assist in classifying a disease or disorder. The classification depends on the formation of a hyperplane that divides binary classes. The SVM locates the hyperplane with the help of the kernel function. A most important advantage of using SVM is to tackle the outliers (Brown et al., 2000). KNN works on the assumption that the instances within a dataset will be close to one another. Although KNN is easy to understand and implement the algorithm, it lacks the fundamental principle in choosing the value of k. Also, it is sensitive to the distance or similarity function used. Decision Tree is made up of nodes and branches, used mainly because of their effectiveness and speed in calculations. Decision Trees are highly prone to overfitting and underfitting of the data (Czajkowski and Kretowski, 2019). Random Forests are the ensemble of Decision Tree. Naïve Bayes is the statistical classification model. Based on the Bayes Theorem, it works on the assumption that all the features in the dataset are independent and equal.

In general, for continuous and multi-dimensional features, neural networks and SVM show better performance. Whereas, in the case of the categorical or discrete features, the logic-based algorithms, such as the rule learners and decision trees, perform better. SVM and others will need a large sample size to produce high accuracy, but Naïve Bayes works on a small dataset. The training time varies for each algorithm; for example, Naïve Bayes trains quickly because of their single pass of the entries. Also, it does not need much storage space during training and testing. On the contrary, during training, KNN based models require huge storage space and more than that during the testing phase.

In terms of interpretability, the logic-based models are interpreted easily, whereas SVM and neural networks are difficult to interpret. They also have the highest number of parameters, which need optimization and tuning. One algorithm cannot outperform the other. One way to determine the type of algorithm to use is to validate the models and estimate their accuracy and choose the one with better accuracy. Recently, combining the algorithms are proposed to enhance individual algorithm performances. However, the gene expression data has the issue of High Dimension and Low Sample Size (HDLSS), for which machine learning models are less suited. Hence, the Deep Learning and Deep Belief Networks are being researched in recent days and a multi-omics dataset.

In the performance evaluation metrics, the commonly used ones are the Classification Accuracy, Least One Out Cross Validation (LOOCV), k-Fold Cross-Validation, and ROC. Among these, several works use the Classification Accuracy. However, many performance metrics need concentration, such as sensitivity, sensibility, and similarity measures.

## Open Issues in Gene Expression Data

The gene expression is a biological process; DNA instructions are transformed into a functional product called the proteins. The cells in a living organism do not need proteins all the time. Certain complex molecular mechanisms must turn the genes on and off. If that does not happen, diseases and disorders will follow.

Deoxy-ribonucleic Acid Microarray is a technology used widely in biomedical research to analyze gene expression to discover the disease or disorder, classify, and predict. The DNA microarray data is also used to predict the responses of a drug or therapies given. There are different types of DNA microarray, such as cDNA (complementary Deoxy-Ribose Nucleic Acid), SNP (Single Nucleotide Polymorphism), and CNV (Copy Number Validation) microarrays (Arevalillo and Navarro, 2013). cDNA is a DNA without introns and formed from a single-stranded RNA. SNP is the variations that can be found only at a single point in a DNA sequence. CNV is a condition where parts of a genome will be repeated, and the repetition will vary from one individual to another. There are many advanced technologies available to analyze gene expression. Most widely used are cDNA bi-color glass slide and Affymetrix GeneChip.

Many challenges and limitations need to be addressed to extract the required knowledge from the gene expression with great precision. The significant difficulties are as follows (Chan et al., 2016; Li and Wang, 2016; Li et al., 2018):

(a) Curse of Dimensionality: The major issue that is researched upon in machine learning is the overfitting of a learning model. The work in García et al. (2017) discusses the curse of dimensionality in detail. Microarray is generally high-dimensional data, ranging from hundreds to thousands and more features. Microarray data prove to be hectic in managing. To handle such huge volumes of data, advanced storage systems are required (Mramor et al., 2005; Abdulla and Khasawneh, 2020).
(b) The gap between the Researchers and Biologists: There is a huge gap among the researchers, biologists and medical practitioners, which led to many unexplored areas in the genomic studies. The opportunity of finding the best techniques and approaches are very less because of the aforementioned gap.
(c) Redundant and Mislabelled Data: Data imbalance and mislabelled data is the most prevailing issue in the Microarray data because of the irregular scanning. The Microarray dataset usually has class imbalance issue, i.e., one class will dominate the entire dataset. When the learning model is trained on a mislabelled and imbalanced data, it will greatly affect the generalization ability of the learning model. Same as the abovementioned issues, redundant and irrelevant data are also the main concern in determining the efficiency of the feature set (Lakshmanan and Jenitha, 2020; Rouhi and Nezamabadi-Pour, 2020).
(d) Difficulty in Retrieving the Biological Information: There are many clinical challenges in retrieving the biological information. The main aim of genomic studies is to discover the significant changes in the gene expression, clinically or biologically. The difficulty is that not everyone will possess high-ended equipment to capture significant changes. Also, in some of the biological processes, the changes in the expression are very subtle and difficult to be identified with analytical methods. Due to the different range of approaches regarding the experimental design, data access, study and batch of reagents used, the data may be erroneous and biased.

Some of the future directions with which the research in this area can be proceeded are as follows:

**(a) Enhanced Models for Better Diagnosis of Rare Genetic Disorders:**

There are various genetic disorders classified under Monogenic and Polygenic disorders. Monogenic disorders are caused because of modifications in a single gene and inherited genetically. It is rare. Unlike Monogenic, Polygenic are commonly occurring and caused because of modifications in several genes. The genetic illnesses of such types are overwhelming in the recent years. Machine Learning classification and prediction models will diagnose the disorders with great accuracy.

**(b) Cancer Prognosis and Prediction:**

Cancer is a heterogeneous disease, which is considered to have various subtypes. It is critical to diagnose early to further assist the patients clinically. The importance of grouping high and low risk patients had led to various researches in bioinformatics and machine learning applications. The ability of machine learning models such as Support Vector Machine (SVM), Artificial Neural Networks (ANN) and Bayesian Networks (BN) in the development of classification and predictive models for accurate decisions have to be explored.

**(c) Collaborative Platforms in Gene Expressions:**

The individual models in Machine Learning will yield better results when applied on gene expression data. However, hybrid methods prove to be successful at many instances. Along with hybrid methods, more research should be done in combining different gene expression data and clinical reports. It is difficult and exhaustive, yet it will offer greater results.

**(d) Analyzing Drug Response in Gene Expression Data:**

Predicting a drug response to any genetic disorder or disease is an important step. Many recent efforts in analyzing the sensitivity and response to cancer or other diseases are commendable. Still, the main problem in developing a model for drug response is the high dimension and less sample size. The feature selection techniques in Machine Learning assist in reducing the dimensions and improve the accuracy in predicting the drug response.

## Conclusion

Gene expression Microarray is a high-dimensional database with less sample size. It needs powerful techniques to handle it and preserve the informative genes by minimizing the redundancy and dependency. This paper discusses the works done in the recent years in the gene expression microarray dataset. The papers are selected from the past six years, the focus is mainly on the supervised, unsupervised and semi-supervised based feature selection in the gene expression data. Further, under those three learning methods, we have chosen papers that concentrate on filter, wrapper, hybrid, embedded and ensemble based gene selection. This study lists out the significant difficulties faced in handling such huge dimensional datasets. To overcome the dimension issues, the gene selection must be made carefully. Although there are a lot of works done in the literature on the gene expression microarray data, there are many open opportunities that need attention. The researches have mainly focused on supervised gene selection with a filter as evaluation methods. The potentials of unsupervised and semi-supervised techniques are yet to be tapped. The semi-supervised technique works with the benefits of supervised and unsupervised techniques combined. Hence, the chances of improved accuracy is high in semi-supervised. The only aim of almost all the works is to achieve higher accuracy the focus on sensitivity, specificity, stability and similarity is scarce. As equally important as the dimensionality issue is the misclassification or mislabelled data. There is a promising future for overcoming these two issues. Another important direction for improvement in gene selection is to develop more ensemble and hybrid evaluation methods. As discussed in the literature, works on hybrid and ensemble are considerably less when compared to filter and wrapper approaches. Hybrid and ensemble methods are capable of providing more accurate results. Apparently, it needs further developments. Research must be done in joint analysis, to combine the clinical reports and the gene expression data. It will help in analyzing various aspects and will offer a different perspective. It would serve as a major breakthrough, yet hectic and exhaustive.

## Author Contributions

PDRV and C-YC did the conceptualization and supervised the data. C-YC carried out the funding acquisition. NM, PDRV, KS, and C-YC investigated the data and performed the methodology. C-YC and KS carried out the project administration and validated the data. NM, PDRV, and KS wrote, reviewed, and edited the manuscript. All authors contributed to the article and approved the submitted version.

## Conflict of Interest

The authors declare that the research was conducted in the absence of any commercial or financial relationships that could be construed as a potential conflict of interest.
